# Pathology

**Published:** 1826-01

**Authors:** 


### PATHOLOGY.

Actio læsa suum designat singula morbum;

Præthereà morbum partis mutatio signat.

1. *New Doctrine of Mental Maladies.** M. Bayle commences, as usual, with reflections on the history of mental maladies, beginning before Hippocrates, and penetrating into the dark æras of mythology, when madness was considered to be owing to a malignant spirit who took possession of the unfortunate individual. Hippocrates dissipated this illusion—attributing insanity to natural causes—especially to black-bile, hot blood, and viscid phlegm. Galen, on the other hand, taught what is the distinguishing doctrine of the phrenologists of the present day, and which is so much cavilled at, *that the brain was divided into a number of organs, each of which had its particular function.* So far he anticipated phrenology. To account for insanity he made some of the four humours accumulate in one or more of the organs or departments, and thus produce different kinds of mental derangement. This doctrine was maintained for some centuries in the schools, with certain modifications. After the humoral pathology lost its vogue, the solidists tried their hands at the pathology of insanity, but with not much better

* Nouvelle Doctrine des Maladies Mentales. Par M. Bayle. Revue Medicale. Fevrier 1825.

success. More lately, Bonetus, Morgagni, Meckel, and others, have sought for the causes of insanity in organic changes in the brain. But they generalized rather too freely, and often put down as a cause, what was merely a concomitant, or even a consequence of the disease. Thus Meckel examined the brains of fifteen maniacs, and found them harder, and of less specific gravity than sound brains. Hence he concluded that a kind of *dryness* of the cerebral mass was the cause of insanity. There are others, among them the celebrated Esquirol, who think that insanity is often dependent on derangement of the vital powers (lesion des forces vitales) of the brain, and sometimes on disturbance merely of the "foci of sensibility" (foyers de sensibilité) in various other parts of the body. Pinel, indeed, thinks that, in the stomach, will generally be found the cause of insanity. But it would be endless to enumerate the opinions which have been broached on the subject. The latest writer on the Continent (M. Georget) comes to this conclusion—"that insanity is always an *idiopathic cerebral affection, the nature of which is unknown.*" He thinks, at the same time, that the organic lesions discovered in the heads of the insane, are consequences, not causes of the mental derangement.

Our present author thinks it remarkable that, while pathologists were searching for alterations of structure in the brain itself, as the cause of insanity, they should have directed so little of their attention to its envelopes—which, however, they have incidently, as it were, described, as very generally changed from the healthy condition. Thus, Morgagni, Meckel, Haslam, Frank, Esquirol have, almost always, found, (independent of cerebral lesions of various kinds,) unequivocal traces of latent arachnitis, or chronic meningitis, as injection and thickening of the pia mater and arachnoid—adhesions of these tissues to one another—collections of serosity on the surface of the brain, or in the ventricles, infiltrations of the pia mater, &c. Why then, asks our author, have not these celebrated pathologists come to the conclusion that chronic inflammation of the meninges was the cause of mental alienation?—Three reasons, he thinks, may be assigned for this. The *first* is, that the brain being the material organ of thought, nothing was more natural for them than to look for alterations of *its* structure, as the cause of mental aberrations. The second reason he assigns is, that they had not a sufficient number of insane patients to form a general doctrine respecting this disease. But the main reason, he conceives, is the want of proper method in studying the malady—namely, by watching the symptoms of each individual case, and carefully comparing them with the appearances on the dissection of these same individuals. Instead of such a method, they observed, as it were on the mass of insane, and generalised accordingly. Thus they observed the various symptoms, in a collection of maniacs—and they recorded the equally various appearances which the brains of maniacs presented—but they did not particularize and connect the individual symptoms with the individual dissections.

In the following Memoir our author lays down his principles and refers to a large work, on the eve of publication, for the facts or cases on which his inferences are grounded.

*Doctrine of Mental Alienation.* The proximate cause of the different species of insanity is not always one and the same, as many authors would fain believe. Sometimes (but more rarely) it consists in derangement of the moral affections—in fact, in *disorder of the mind or soul*, in which class is always to be found monomania, or insanity on a single point, as also melancholia. This species might be designated or defined a "reigning error," (erreur dominante) which holds in subjection, more or less, the will of the individual. In the great majority of cases of insanity, however, the disease is produced by a physical lesion, consisting almost always of chronic inflammation of the meninges (arachnoid and pia mater)—and sometimes in a specific or sympathetic irritation of the brain. Here, of course, our author does not speak of idiotism, produced by congenital malformation or organization of the brain.

This chronic inflammation presents two species. Sometimes it is seated in the arachnoid, or even in the arachnoidean sheet, which is reflected over the inner surface of the dura mater:—Sometimes it commences in the pia mater, which becomes more or less injected, and thence spreads to the tunica arachnoidea. In these cases, the lining membrane of the ventricles (l'arachnoide ventriculaire) is generally affected. The first species he denominates "chronic arachnitis," because its seat is generally in the tunica arachnoidea, and it is often slight in degree:—the second species he denominates "chronic meningitis," (affecting both tissues) being always of very long duration. In his forthcoming work, M. Bayle hopes to prove, even to demonstration, the truth of this theory of mental alienation. Private motives (the fear, we should suppose, of being anticipated) induce him to publish the principles before the facts on which they rest. These facts, he informs us, consist of two hundred cases, observed with the most careful attention in the MAISON ROYALE DE CHARENTON, one of the most splendid establishments for the insane on the Continent. The observations were made under the eye of Professor Royer Collard, who is much eulogised by M. Bayle, for his talents and acquirements.

The cases of insanity, he observes, which are dependant on chronic meningitis, are very frequent, forming, on an average, one-fifth of the whole number, *in males*—while (which is curious) the proportion is diminished *in females*, to rather less than *one thirtieth*. Into the etiology of meningitis, as the proximate cause of this species of insanity, our author does not enter. But he remarks, en passant, that this *chronic* inflammation of the meninges is rarely or never the sequel of *acute* inflammation of the same parts. It is the result of sanguineous congestion in the vessels of the pia mater, which sometimes comes on suddenly, with loss of sense, redness of the face, or even paralysis—sometimes in a more slow manner, with vertigo, numbnesses, cephalalgia, &c.—and, at other times, in a most latent and insidious form of approach.

1. *Anatomical Character of Chronic Meningitis.* When the pia mater is the seat of this phlogosis, that membrane becomes red and injected—the arachnoid thickens, losing part or the whole of its transparency—becomes more tenacious—exhales more or less of fluid—contracts adhesions with the pia mater or dura mater—becomes covered, in conjunction with the pia mater, with granulations, exudations, false membranes, &c. Some of these appearances are constant—others only occur under certain circumstances.

*Seat of these Lesions.* These lesions always occupy those portions of the arachnoid and pia mater which cover the convexity and internal face of the cerebral hemispheres. That portion of these membranes which is spread over the base of the brain and the cerebellum is always sound. The ventricular arachnoid is often affected.

*Injection of the Pia Mater.* In the majority of cases, the pia mater is red and injected. Its vessels are often so dilated, that the tissue appears thickened, and when it is torn up from the brain below, a quantity of blood flows out, more or less mixed with serosity.

*Thickening of the Arachnoid.* This is one of the most constant characters of chronic meningitis; but it is extremely various in degree. Our author has seen it as thick as the pleura, pericardium, dura mater—nay, even as substantial as the parietes of the stomach. In these cases it often assumes the appearance of parchment softened in water. In many instances the ventricular arachnoid participates in the thickening. A corresponding degree of opacity necessarily accompanies the thickening of this tissue. The same may be said as to its density.

*Serous Effusion.* When the arachnoid is affected with chronic inflammation, it exhales, as other serous membranes do, a quantity, more or less, of serosity. The seat of this effusion is generally between that sheet of the arachnoid which is spread over the pia mater and that which is reflected over the internal surface of the dura mater. It is most abundant about the basis cranii, where it is found sometimes to the amount of several ounces. A quantity will also be found to issue from the spinal canal. Our author once found 12 ounces of this effusion on the superior surface of the brain, the convolutions of which had been much flattened thereby, and the dura mater much distended. The lateral ventricles also will be found to contain more or less of serous effusion.

*Infiltration of the Pia Mater*, is another constant character of chronic meningitis. It is always to be found opposite to those parts that are affected in the arachnoid. The fluid is contained between the laminæ of this membrane and is with difficulty estimated as to quantity. It is generally considerable.

*Adhesions of the Meninges.* These are not unfrequent in chronic phlegmasiæ of the parts. The arachnoid and pia mater are not only adherent, here and there, to each other, but also to the surface of the brain, from which they cannot be separated but by force. The portions of the brain torn up, when these adhesions are raised, are softer than natural. These adhesions are almost exclusively confined to the external surfaces of the convolutions, and are rarely found in the anfractuosities or clefts between the said convolutions.

*Granulations of the Arachnoid.* These are often seen, in the form of very minute spherical bodies, similar to those which we see on the surfaces of other serous membranes, when affected with chronic inflammation. These granulations, of infinite minuteness, are often found in great numbers, on the internal surfaces of the lateral ventricles. It sometimes requires the aid of glasses to detect them.

*Sanguineous and Albuminous Exhalations—False Membranes.* Sanguineous effusion is seldom seen in the arachnoid cavity, except where there are also false membranes. These membraniform exudations, our author avers, are found in about a sixth or seventh of those who die of chronic meningitis. "Their seat is always between the two sheets or laminæ of the arachnoid—in the cavity of that membrane."* They are seen spreading over the convexity of one or both hemispheres, and extend more or less towards the base of the brain, which they sometimes cover. These false membranes are sometimes transparent, when they are very thin; but mostly they are of a whitish or yellowish colour, and opake. These false membranes sometimes acquire an extraordinary degree of thickness—even to that of two lines. They are often accompanied by sanguineous effusion, in the form of blackish clots, of greater or less extent, situated between the laminæ abovementioned of the tunica arachnoidea, and generally on the upper part of the brain.

Such are the lesions found in cases of *chronic* meningitis, and it must be acknowledged that they bear considerable analogy to those observed in the *acute* inflammations of the same parts. Our author has laid down some distinctions between the post mortem appearances in the acute and chronic forms of the disease, but we think they are not very well defined, nor do we see any great utility in the distinctions. The history and symptoms of the disease will best enable us to say whether the disease is acute or chronic.

*Symptoms of Chronic Meningitis.* These are very various and complicated, as may be readily supposed, from the great importance of the parts affected. In order to present a portrait, at once faithful and succinct, of the phenomena attending this disease, our author thinks

* His meaning is, between the tunica arachnoidea, as we commonly see it, and that layer of it which is supposed to be reflected over the inner surface of the dura mater.—*Ed.*

it necessary to divide the period of its duration into three epochs or stages.

1. *First Period; or Stage of Monomania.* The first symptoms, according to our author, often manifest themselves immediately or a few days after an attack of cerebral congestion. The patient has experienced vertigo, numbness, or some local or general loss of sense, with more or less of paralysis—at other times, the invasion of the disease is not preceded by any of these phenomena. The individual imagines himself all at once to be possessed of riches, power, rank, dignities, or other distinctions. Under the dominion of some idea of this kind, he can neither talk nor think of any thing else—and, if crossed in his opinions or conversations, he gets into a rage. Nevertheless, if questioned on any subject unconnected with their hallucination, these individuals will answer with sufficient rationality. If examined more closely, it will be perceived that several of their intellectual faculties are much weakened. Their attention and their memory will be found defective—and they are incapable of attending to their habitual occupations.

In the mean time, remarks our author, there will be observed, in almost every instance, a certain degree of embarrassment in speaking—that, is, a certain loss of power in the tongue. This defect will not, indeed, appear during a state of excitement, but will be readily recognized when they are tranquil. Not unfrequently, with this lingual embarrassment will be joined a certain degree of defect in the lower extremities, as evinced by some irregularity or halt in their walking. This phenomenon, however, is by no means constant.

*Second Period.* This period is divided into degrees, and subdivided into varieties, by our author, with a great deal too much of subtlety for practical purposes. After all, this second period differs only in degree from the first period— that is, there is an augmentation of the symptoms —" il consiste uniquement dans une augmentation des symptomes." The hallucination or delirium becomes more general, but still with a predominance of the monomaniac illusory idea. " Les malades sont dominés par les mêmes idées que dans la première periode, mais le delire est général." The intellectual faculties are quite in disorder—and the defect of muscular power is also much more considerable than in the first period. They now become inattentive to every thing passing around them, being entirely occupied within themselves. And here we do not think it necessary to copy our author's minute descriptions of the ravings of a maniac. He might just as well attempt to pourtray the ever changing and unmeaning forms of the clouds that pass over his head. It may nevertheless be amusing to our readers just to exhibit a single passage of M. Bayle's symptomatology, which he seeems to think so very interesting as to mark it in Italics. Speaking of certain descriptions of the insane, he says—" *Ils possèdent des millions et des milliards—ils sont princes, rois, empereurs—ils font cent lieues en un jour —ils ont cassé le pont qui va à la lune—ils ont le pouvoir de resusciter—*

*ils ont la flamme et les eclairs dans les yeux—ils se grandissent à la volonté—ils ont la tête d'or et de diamant—ils font cent tragedies superbes en un jour, mille poëmes—ils ont tout fait—tout leur appartient, &c. &c."* Now, it would have been quite as much to the purpose, and have saved a multiplicity of words, if M. Bayle had said at once—" an insane person talks all kinds of nonsense." It is really a species of insanity to record their extravagant expressions.

It is more to the purpose when he describes phenomena in the following manner:—The insane, in this second period of meningitis, when questioned as to their profession, age, &c. either make no reply, or utter absurdities. They are in constant agitation, talking incessantly, and with great volubility—they are ever in motion, and incapable of remaining a minute in one place. Led, as it were, by some irresistible impulse, they know not what they do nor where they go. In this condition, they are apt to do mischief to themselves or the things around them; and, therefore, the straight-waistcoat becomes necessary. In such a state of excitation, all symptoms of paralysis disappear. It is only necessary to add, that not very unfrequently we see the maniacal paroxysm become periodical, returning at regular or irregular intervals—sometimes every day—more frequently every other day.

*Third Period—or Dementia.* This period is characterised by a kind of mental collapse or exhaustion, somewhat analogous to that which takes place in the corporeal fabric after a period of much excitement. The intellectual faculties become much enfeebled, with a greater or less degree of obliteration of ideas—but still with a predominance of the idea which first appeared in the monomaniac form. This stage or period is generally accompanied by paralysis in some part or other, not unfrequently with convulsive movements, or epileptic or apoplectic attacks. This third period is again subdivided by our author into three degrees—and M. Bayle becomes excessively tiresome by his endless descriptions. It is sufficient to say that, in the worst degree or ulterior stages, a state of stupidity takes place, with considerable paralysis. The patient is reduced to a dreadfully low ebb in his moral and physical condition—far, indeed, below that of the brute creation.

*Connexion of the Symptoms with the Organic Lesions.* The following are the corollaries drawn from the attentive examination of two hundred cases, with their dissections.

1. Chronic meningitis is the proximate cause of one fifth of mental diseases in men, and of from one thirtieth to one 35th, in women.

2. It is produced generally by sanguineous congestion, sudden or slow, in the vessels of the pia mater.

3. It commences on the internal surface of the arachnoid, whence it spreads to the rest of the membrane—always, however, confined to the convexity and internal face of the hemispheres, and to the ventricles.

4. It commonly presents three periods, to wit—one of sanguineous congestion of the pia mater, with irritation of the tunica arachnoidea—

one of inflammation of this membrane—and one of exhalation, each of which gives rise to a mental alienation or disorder, corresponding with these three periods—viz. monomania, mania, and dementia.

5. The delirium, in this disease, always depends on the irritation which the inflamed pia mater and arachnoid propagate to the cortical substance of the brain.

6. The *monomania ambitiosa* of the first period, and the ideas of grandeur, power, and opulence which we observe in the individual, coincide always with a durable sanguineous congestion (avec une congestion sanguine durable) in the vessels of the pia mater, accompanied by irritation of the internal surface of the arachnoid.

7. The slight traces of incomplete paralysis, in the first period, indicate a compression of the brain produced by sanguineous congestion.

8. The exaltation and restlessness of this period are produced by the secondary excitement of the brain, irritated by the internal surface of the arachnoid spread over it.

9. The general delirium and agitation, more or less violent, which accompany it, and which are observed in the second period, indicate that the cerebral irritation and arachnoid inflammation, on which they depend, are very considerable.

10. The agitation, so violent and constant, is often occasioned by a very intense inflammatory process, which gives way to an albuminous exhalation on the surface of the arachnoid.

11. The blind and incoercible spasmodic agitation, coming on in quotidian or tertian accessions, as also the epileptic attacks, depend on consecutive inflammation of the brain, the surface of which becomes softened, and contracts adhesions with the pia mater and arachnoid.

12. The tremors, partial or general, the subsultus tendinum, the convulsions, grindings of the teeth, rigidities, contractions, &c. depend also on consecutive inflammation of the cortical substance of the brain.

13. The apoplectic attacks, so frequent in the third period of insanity, are almost always owing to sudden sanguineous congestion in the vessels of the pia mater and brain—rarely to serous effusion—and never to hæmorrhage.

14. The cessation or diminution of the agitation—the weakening of the intellectual faculties—the paralyses which we observe in the latter stages, are the signs of cerebral compression, depending on exhalation of serous fluids into the cavity of the arachnoid, infiltration of the pia mater, and effusion into the ventricles.

15. The increase of the paralysis, and of the dementia, indicates a corresponding increase of the cerebral compression.

16. The state of stupidity, with obliteration of ideas and the more or less complete paralysis, is the consequence of compression of the brain, the result of serous effusion carried to the highest degree.

Such are the corollaries which our author has drawn from his observation of symptoms and dissections. We confess we cannot see any just cause why M. Bayle should have headed his paper with the dangerous title "*nouvelle doctrine*" des maladies mentales. If this *new*

*doctrine* consists in tracing mental alienation to disease of the brain or its membranes, he has been anticipated by hundreds—for instance, by his own countryman, Georget, whom he cites, and who thinks that insanity—" est toujours une *affection cerebrale idiopathique.*" But how does M. Bayle prove insanity to be *caused* by meningitis? By shewing that meningitis takes place in nearly *three cases* in every hundred instances of insanity—allowing that the patients are females! In short, our author contradicts himself in this paper. He says that " sometimes, but not frequently, the proximate cause of insanity consists in a lesion of the *moral affections*"—a malady of the soul—" en une maladie de l'âme." And again, " in the greater number of cases, mental alienation is caused by a physical lesion, qui consiste, *presque toujours*, dans une phlegmasie chronique des meninges." Yet, when we come to facts, this " *presque toujours*"—this " almost always," dwindles down to one fifth in men, and one *thirty-fifth* in women!! Now, we believe (as we are in duty bound) M. Bayle's facts;—but we demur to his inferences. We believe that in some such proportion as he has stated, we shall find meningitis—and, in such proportion, we have no objection to consider this lesion as the cause of the phenomena. But when we find that four out of five in men, and 34 out of 35 in women, present no such lesion, according to his own shewing, we certainly shall not come to his conclusion, that mental alienation is *almost always* caused by chronic meningitis. On the contrary, we should be inclined to conclude that *moral causes* produce insanity, in the majority of cases, by disordering the intellectual *functions*—and that the physical lesion, *meningitis*, is but rarely (comparatively speaking) the cause of the mental malady. Finally, while we attach importance to the *pathological facts* which our author has collected, we give but slender credence to his theory—and utterly deny its novelty—excepting that part of it which makes "*presque toujours*" mean a *thirty-fifth.*

---

2. *Phrenitis—Arteritis.* [Mr. Howel, Med. Repos., Sept. 1825.] On the 11th April, Mr. H. was summoned to a gentleman, 28 years of age, a painter, of steady and temperate habits, who had been suffering pain in his head for some days. Leeches had been applied and purgatives administered. He now complained of acute darting pain in the head, with full, hard, and quick pulse. Tongue loaded and breath offensive—temperature of skin not augmented—no intolerantia lucis. *Venesection to sixteen ounces, which produced syncope. Calomel and salts, with senna. Head to be shaved and kept wet.* In the evening the head-ache was much increased—the pulse hard and full, at 120. Yet the blood drawn in the morning had no appearance of inflammation. *Bled again to syncope—applied* 12 *leeches to the head—a blister to the occiput.* 12th. The symptoms rather mitigated—no inflammation in the blood drawn. His bowels are free, and he voids a great deal of *pale* urine. *A saline mixture, with digitalis.* In the evening he had a cool, moist skin, and the *pale* urine still more copious. 13th. Passed an easier night; but the pain of head is increased this morning. *Leeches to be repeated, with blisters behind the ears—calomel and antimony at bed-time.* 14th. Has passed a restless night—head-ache much increased—skin hot and dry—pulse hard, full, 120—bowels freely opened. *Venesection to 20 ounces with complete syncope.* Was seen by a physician at noon, who was struck with the immense volume and force of the pulse. The action of the heart was distinctly audible in any part of the bed-chamber. Yet the blood taken in the morning had no appearance of inflammation—it was loosely coagulated. *Digitalis increased in dose—calomel at bed-time.* In the evening the pain of head had increased much—the countenance was pale and anxious. *Venesection to sixteen ounces*—syncope. 15th. Head easier—pulse reduced—complains of slight sore throat, with short dry cough. 16th. Pain in the head as violent as ever—distressed with incessant hiccup—no pain in the chest. The pulse being full and quick, 20 *ounces of blood were abstracted from the temporal artery*, which produced fainting. The hiccup is still constant. Bowels regular and evacuations healthy. Is now taking half-drachm doses of the tincture of digitalis, without any apparent effect. A blister to the scrobiculus cordis. 17th. Head relieved—pulse reduced—hiccup continues. 18th. Head free from pain—hiccup continues—complains of pain in the chest—pulse hard and full—action of the heart violent. The depleting plan was objected to by the friends. An opiate was ventured on for the first time. 19th. Passed a disturbed night—hiccup continues—no head-ache—pulse full, hard, 120. An erysipelas on the right side of the head and face. *An enema of tobacco infusion produced no effect. Is taking* 40 *minims of tincture of digitalis and* 30 *of hyosciamus every four hours.* 20th. The heart is acting as violently as ever, "the arteries feeling as if they would burst under the pressure of the finger." Complains of no pain. The hiccup continues. *Nauseating doses of antimony.* These had no effect on the pulse, and were discontinued. *The chest to be rubbed with antimonial ointment.* 21st. Had some sleep—hiccup less—slight diarrhœa—pulse unaltered. *Blister to the chest. Evening.* Hiccup as violent as ever. Mr. Callaway saw him this day, and was astonished at the tremendous pulse. *Venesection to* 20 *ounces*, and the blood was now, for the first time, remarkably cupped and buffed. He became extremely faint, the hiccup ceased, and he slept for an hour. He then returned to his former condition. *He was ordered the liq. plumb. subacet. with tinct. opii, every four hours.* 22nd. No material alteration in the symptoms—countenance worse—face swelled, and of a dark purple hue—tongue dry and brown. *Venesection to* 16 *ounces.* Blood strongly inflamed. Two grains of calomel and one of opium every three hours. In the evening he became delirious, and next day (23rd) he died in a convulsive paroxysm.

*Dissection. Present, Mr. Howel, Mr. Callaway, and Mr. Ruris.* The brain was remarkably firm; and, upon being cut into, numerous red points were observable. "With the exception of this, there was no other diseased appearance in the cavity of the cranium." "All the viscera of the thorax and abdomen were quite healthy; and no mark of disease could be discovered till the pulmonary artery was slit open: the inner coat of this vessel was found in a highly inflamed state, being beautifully injected, from the semilunar valves to its division into right and left; the inflammation had not extended into the ventricle, nor into either of the divisions of the artery. The heart itself, the venæ cavæ, and all the neighbouring parts, did not present the slightest vestige of disease."

*Remarks.* This is a very interesting case, but not without several anomalies and difficulties, which are not cleared up by dissection. We much doubt whether the narrator is justified in designating it "a case of phrenitis, *followed* by arteritis." The symptoms during life do not authorise this conclusion. There was dreadful pain in the head; but excessive pain is rarely complained of in real inflammation of the brain. There was no intolerance of light—no delirium (till near death)—no marks of inflammation in the blood (for many days at least)—no redness of the eyes or face, but the contrary—and, finally, there was a copious discharge of *pale urine*. This last symptom alone would almost convince us that acute inflammation of the brain, or of any important organ, did not exist contemporaneously with it—at least, in the course of 30 years' observation, we never saw pale and copious urine attend acute inflammation of an internal organ. The *post-mortem* appearances in the brain are far from being proofs of inflammation. Numerous red points prove, we grant, "distention of the minute arteries"—but distention and inflammation are far from synonymous. We see these red points in many cases where there is no inflammation, but merely congestion of vessels. Again, can our author suppose that *firmness* of the brain is a token of inflammation? Let him peruse the late pathological investigations of diseases of the brain, especially those of Rostan, Bricheteau, Lallemand, Abercrombie, &c. and he will see that inflammation produces the reverse of firmness in the encephalic mass. Our conclusion then is, that inflammation of the pulmonary artery was the original and sole disease:—that from this sprung the tremendous action of the heart—and that from this tremendous and indomitable action of the heart arose the insufferable head-ache and the distention of the capillaries of the brain. Over this peculiar and fatal inflammation, we appear to have little power. It is abundantly evident that all the depletion used in the above case was productive of little, if any benefit. In short, we know not, at present, how to reduce arteritis. Its symptoms, we see, are not those of common inflammation of a vital organ, witness the anomalies pointed out:—it is not amenable to the same laws—it is not, unfortunately, governable by the same remedies. It is of some consequence, however, to know when we have so formidable an enemy to cope with, and the case related is important in this point of view. We think it will be granted that the head engrossed too much of the attention, in the case under review—and that, upon a future occasion, such symptoms will lead to a clearer diagnosis. We do not, indeed, mean to say that any other treatment would have been successful; but we think the true nature of the complaint was overlooked, at least in the beginning; and that the title of the case involves an erroneous conclusion.

---

3. *Pulmonary Phthisis cured, &c.* [*Archives, Mars,* 1825.] The nature of this case, and the object of its publication, will be best understood by the narrative itself.

M. D. a painter, aged 27 years, with narrow chest, pale complexion, and born of phthisical parents, had been affected, for two years past, with a short dry cough, to which he paid little attention. He had also felt some pains in different parts of the chest, especially under the clavicles. In December, 1823, he was seized with a bowel-complaint, and passed a considerable quantity of black blood, followed by extreme debility. The diarrhœa sanguinolenta was removed by a decoction of rhatany, with some other simple prescriptions, but a great degree of emaciation was the consequence. He had begun to convalesce, when, in January, 1824, some blood appeared in his expectoration. On the 17th, the thorax was examined by the stethoscope throughout, and under the *right* clavicle was discovered a space presenting the "*râle crepitant,*" well marked. On the 19th, in the evening, there was a febrile accession, and, in the night, the cough was very troublesome—towards morning, perspiration. These phenomena took place on the succeeding nights. 23rd. A Burgundy-pitch plaster, with tartar emetic, was applied to each side of the chest. The emaciation went on. 28th. A blister to the place under the clavicle, whence the crepitous rattle had been heard. The next night, after a smart paroxysm of fever, there burst forth, all at once, a considerable quantity of heavy purulent expectoration, which sunk in water, and contained shreds of membrane and other debris. The stethoscope, now applied under the clavicle, discovered unequivocal pectoriloquism in the spot beforementioned. 30th. In a consultation, it was determined to make two caustic issues over the part where the excavation was situated. These were effected on the 1st and 5th of February. The eschars were large and deep. Other counter-irritation was also kept up on the parietes of the chest. Slight amelioration of the symptoms after the first caustic; afterwards, the nocturnal fever and perspiration gradually diminished, with some return of appetite. Flesh and strength progressively increased; but the purulent expectoration still continued, and the pectoriloquism remained audible. The amelioration of symptoms continued a few days, and then the night-sweats returned. On the nights of the 17th and 18th of February they were very considerable, and, on the 19th, blood shewed itself again in the expectoration. The strength now rapidly declined—the pulse became quick and hard—the appetite disappeared—and the patient lost his courage. Exploration of the chest now discovered, under the *left* clavicle, a small space exhibiting the "râle crepitant." The night-sweats and perspiration daily increased. Pectoriloquism was soon afterwards manifest, though to a very small extent, in the space abovementioned, under the *right* clavicle. The diet was still farther lowered. Decoction of Lichen Islandicus was prescribed. A caustic issue was applied to that side, soon after which the symptoms began to abate—the countenance improved—the strength increased—the pulse fell to 68 or 70—the expectoration decreased—the appetite became keen. Farinaceous food was given and augmented gradually in quantity. Nutrition soon became apparent, and the patient was able, in a short time afterwards, to work a little at his occupation. Every thing, in short, announced convalescence. Some of the issues were allowed to heal, and the others lessened in size.—Fish was added to his farinaceæ and milk—then poultry—and ultimately flesh-meat. Examined frequently by the stethoscope, this man exhibited the pectoriloquism on both sides. Between the 15th and 20th March he had two or three febrile accessions, with night-sweats, and increase of expectoration, without any apparent cause. These phenomena were attributed to the breaking down of some small tubercles into the existing excavations. Half a grain of the superacetate of lead, night and morning, diminished the expectoration and hectic perspirations. By the 20th March the pectoriloquism could no longer be heard on the left side. By the 1st April, the cough and expectoration ceased rather suddenly; and yet pectoriloquism was as evident under the *right* clavicle as on the first day of its appearance. The health now improved daily. The patient could go out and take pretty long walks. By the end of April the pectoriloquism was more circumscribed than hitherto; and at this time he caught a cold, and exhibited symptoms of slight bronchitis, which were soon dissipated by proper means. He was recommended to go to the South of France, which he did in the beginning of May, and placed himself under the care of Professor Lallemand. The latter gentleman wrote to Dr. Roche, under date of July 5th, that the patient had been troubled with profuse night-sweats soon after his arrival at Montpellier, but that they had then entirely disappeared. He scarcely coughed or expectorated—his strength was nearly restored—and the space of pectoriloquism was diminished one third.

From the above circumstances, M. Roche comes to the conclusion, and apparently with reason, that the patient had had, for a long time, a conglomeration of tubercles at the upper part of each lung—that these had softened down, suppurated, and been expectorated—that the consequence was, an ulcerated cavity on each side, extensive on the right, circumscribed on the left—and, finally, that these cavities had healed, the one probably by means of a cartilaginous lining, the other (the left) by adhesion. We cannot but come to the same conclusion; and we think our readers will consider the case altogether as curious and interesting.

---

4. *Hospital Reports. La Charité.** The practice of publishing quar-

* Laennec's Wards. First Quarter of 1825. Reported by Meriadec Laennec. Revue Med. June 1825.

terly reports from the Parisian Hospitals goes on successfully; and we hope our hospital physicians, on this side of the water, will at length be roused to such a sense of their duty to the public, as shall induce them to follow the example. The increasing avidity for information—the extension of the press—in short, the temper of the times, demands publicity for all transactions that take place within the walls of our sanctuaries for sickness. The impulse cannot be resisted, though the period of its fulfilment may be procrastinated by timidity, selfishness, or pride. An æra has arrived when the actions of men in public situations—and among others the medical officers of hospitals, must undergo the inspection and submit to the comments of a wider range of critics than the pupil train that were heretofore the sole observers, (for they can hardly be called *judges*,) of the professional practice of the said officers. The practices of public institutions will get abroad, as indeed they ought to get abroad, and if so, we submit it to the prudence of the medical officers themselves, whether it is not better that the reports should be fairly, candidly, and officially made, than that they should be liable to every kind of distortion and falsification, from the ignorance, caprice, or malice of private reporters. The adoption of some plan of this kind is no longer a matter of choice but of necessity. The sooner it is put in execution the better. It is no Utopian proposal. We see it adopted, with the best effects in other countries, and there can be no valid objection against its adoption here.

1. *Fever.* Of 160 admissions into Laennec's wards during the quarter, 83 were acute diseases, and 67 chronic. The mortality was 29, or rather less than a fifth part. Of 23 cases of *severe fever* there were only two deaths—though the treatment was Hippocratic, or *expectante.* The head was the organ that suffered most, in this quarter. Laennec did not confine the fever patients to so rigid a diet, or rather abstinence, as is generally done in such cases. He thinks, that abstemiousness, too long protracted, injures the tone of the stomach, and is one great cause of the inconvenience felt by convalescents when they begin to take nourishing diet. M. Laennec rarely applied leeches, and only when there was acute local pain, or appearance of congestion about the head. He seldom applied blisters or other counter-irritants—and rarely prescribed purgatives, unless there was obstinate constipation. He ordered laxative lavements from time to time. The mortality was certainly very small, especially when it is stated that, of the two who died, one laboured under a tuberculous excavation in the lungs, previously to the accession of fever. The crises of fever have been pretty generally observed by Laennec at the septinary periods—a circumstance attributable to the very little interruption that was offered to the workings of Nature. The report shews that fevers will be cured by very little—indeed, by no medicine, contrary to what is the case where there are phlegmasiæ of the internal organs—accompaniments, but not essential parts of fever.

2. *Acute Rheumatism.* M. Laennec trusts almost exclusively to antimony in large, or Italian doses, for the treatment of this disease. Tartar emetic, from six to ten or twelve grains daily, is given in solution, and undivided doses. It supersedes local and general bleeding.

3. *Colica Pictonum—Peritonitis.* Young Laennec relates, with great surprise, a case which would not cause so much wonder in this country. A young man was under treatment for colica pictonum, but apparently with little success. Meanwhile an unequivocal peritonitis supervened, and the usual treatment of La Charité was instantly suspended. Leeches were applied to the epigastrium, which gave a momentary relief. Large mercurial frictions were quickly employed, (half an ounce of strong mercurial ointment daily,) and on the fifth friction, ptyalism arose, and continued more or less for a month. From the moment the salivation appeared, every other symptom of disease disappeared. "Mais le malade se trouva parfaitement guére, et de la peritonite, et de la colique de Plombe, dès qu'il eut commencé à saliver." It has long been known in this country, that mercurialisation arrests the colica pictonum—and that it is no mean antidote to peritoneal, or indeed any other internal inflammation, whatever may be the scepticism of some practitioners who are blinded by prejudice.—"Ce succés des frictions mercurielles, dans un cas aussi grave, prouve assez combien elles meritent de confiance dans le traitement de la Peritonite."

4. *Phthisis.* Passing over some of M. Laennec's observations on apoplexy, and on the propriety of using large doses of tartarised antimony in such cases, (which we find he does) we come to the subject of phthisis. In this opprobrium medicorum he has given a considerable trial to the hydriodate of potash, in frictions, without any notable advantage or inconvenience. He has also endeavoured to appreciate the value of the ancient opinion (still maintained by some modern physicians, especially in England) relative to the good effects of navigation and sea air in this complaint. Having observed that, on the coast of Britanny, where the air is more humid, but, at the same time, more mild and equable than in the interior of France, the number of phthisical patients was comparatively small—and having also seen that young men from Britanny became consumptive during their sojourn in large cities, and recovered on returning to their native province, exhibiting unequivocal proofs of pulmonary cicatrices, closed or fistulous, (presentant des traces non-équivogues de cicatrices pulmonaires pleines ou fistuleuses) he came to the conclusion that the peculiar atmosphere of the sea-coast had something to do in these results. He, therefore, tried to imitate it, in some measure, by placing near the beds of the patients certain fresh marine plants. He brought together, into two small wards, a number of phthisical persons, and surrounded their beds with the *fucus vesiculosus*, causing them to drink also an infusion of the same plant. None appeared to suffer from this mode of treatment; most of them seemed to be very comfortable in these wards for a space of four months—that is, as long as the fresh fucus could be procured. The cough became less frequent, the breathing less confined—the expectoration in less quantity. In the greater number, the hectic fever ceased, and the progress of the emaciation was arrested. Towards the end of March (1825) five of the patients demanded their discharge, considering themselves as free from disease. Of these, however, there was only one who afforded well grounded hopes of cure, in the eye of the physician. This was a young woman, 24 years of age, who, on entering the hospital, presented unequivocal pectoriloquism under the right clavicle, and appeared to be rapidly sinking. She continued more than three months in a stationary condition in the hospital, and then gradually acquired force and flesh. When she left the hospital the pectoriloquism was no longer audible. In the course of the month of April it became impracticable to procure, or rather to keep the fucus, and, from this time, the march of phthisis acquired great rapidity among the patients in the hospital, and they were all carried off in the course of a month, with the exception of the five abovementioned, who were discharged.

Upon this statement we shall not make many remarks. It appears to us very improbable that the presence of the fucus could have had much effect in the retardation of the disease above alluded to. It was more probably due to the regulated temperature of the wards, or some other circumstance that does not appear on the face of the record.

The chlorate of lime, in vapour, was tried in the phthisical wards by M. Laennec, in consequence of a popular belief that it has a powerful influence in checking consumption, but it had no perceptible effect in this way.

5. *Diseases of the Heart.* Of 18 cases of this kind contained in the table for this quarter, there were four which turned out to be only nervous affections of the organ of circulation. Laennec has been exerting himself in clearing up the diagnosis between real and apparent hypertrophie of the heart. In this last, (ou simple agitation, nerveuse du cœur) says he, the action of the heart is always accelerated without a corresponding febrile movement in the system, and the pulsations of the organ, though strong in appearance, are not accompanied by elevation of the pectoral parietes. In real hypertrophy, on the contrary, the thoracic parietes are actually elevated at each impulse of the heart, and so is the head of the auscultator, when he is examining the heart's action by means of the stethoscope.*

The hissing, murmuring, or purring noise, which is very generally heard when there is disease, especially puckering or stricture of the

* With every deference for the opinions of Laennec, we cannot quite agree in the foregoing diagnosis. We have seen instances of nervous palpitation in females, where the ribs were raised at each stroke, and where the whole bed vibrated with the inordinate action of the organ. In our earlier days we were often led into false prognoses from these phenomena. We have long since learnt to be more cautious.—*Rev.*

orifices of the heart, has been heard in several instances by Laennec, where there was only a nervous affection of the central organ. He attributes the phenomenon to spasm, but he is evidently unable to account for it in any satisfactory manner. It will be prudent, however, for the auscultator to bear in mind that this phenomenon is not an unerring mark of organic disease of the heart, as is very generally supposed by those who profess to study the pathology of this viscus. Laennec properly considers deep-seated pain in the præcordial region as no sure criterion of cardiac disease. This pain often accompanies nervous palpitation from a moral cause. "When this pain is severe and lasts for some time, it generally denotes a neuralgia of the cardiac nerves, in which case there is almost always a sense of constriction at the lower part of the thorax and numbness of the left arm. This is what constitutes the angina pectoris of authors,—an affection which M. Laennec has frequently seen existing without any organic disease of the heart itself." We believe this is the case sometimes. We examined, with the greatest care, a few years ago, a man who long had symptoms of angina pectoris, and who died in one of the attacks; yet we could find no appreciable change of structure in the heart on dissection. It is very rare, however, not to find the pathological condition of the organ in correspondence with the symptoms during life.

6. *Aneurism.* There was a case this quarter of aortic aneurism, situated in the middle of the pectoral portion of the descending aorta. The patient had fixed pain in the left inter-scapular space, sometimes extending, with great severity, to the whole of the left side, following the direction of the intercostal nerves. All that part of the back comprised between the fourth and eighth ribs, emitted a dull sound on percussion, and yet the respiratory murmur was distinctly heard there by the ear or stethoscope, though fainter, and seemingly deeper than elsewhere. This induced Laennec, on the first examination, to affirm that a foreign body, and that a good conducter of sound, was interposed between the lung and the ribs. In a few days more he hesitated whether the foreign body was an aneurism, or a collection of matter with caries of the vertebræ. The patient was now stricken suddenly with incomplete paraplegia, which led M. Laennec to think that the aneurismal sac had opened into the spinal canal. He died in a few hours after this accident. On dissection, a communication, six lines in diameter, was discovered between the aneurism and the vertebral canal. Blood had penetrated and formed a clot sufficient to compress the spinal marrow and produce the paralysis. This last, however, was not the immediate cause of death. The sac had also opened into the cavity of the pleura, which was filled with coagulated blood.

In the treatment of diseases of the circulating organs, Laennec suggests the employment of preparations of lead, in conjunction with the starving and depletory system of Valsalva. He is led to this proposal from observing that, in those who have died of colica pictonum, all the different tissues of the body are exceedingly pale, and, as it were, exsanguineous. If then the preparations of lead have the effect of producing a degree of anemia, they may become important auxiliaries to the treatment of Valsalva. The trials made at the hospital by our author are not yet sufficiently forward for solving this interesting problem.

7. *Abdominal Tumours.* Of three instances of tumour in the abdomen this quarter, two excited some interest. The first was an enormous cyst of the right ovarium, which the patient had borne for 20 years, the contents of which are supposed to be blood extravasated there; but the fluid is under a rigid analysis.

The second case was a medullary or encephaloid tumour, having its seat partly in the ovaries, partly in the parietes of the uterus. The woman said she had been ill only two months :—That her complaint began with an inflammation of the bowels, for which 400 *leeches* had been applied in the course of eight days! It was only since this event that the tumour made its appearance. She was in a state of complete marasmus when she entered the hospital, where she died ten days afterwards. It was natural enough for Laennec to search for traces of the peritonitis which had required *four hundred leeches* for its subjugation. But no trace of peritonitis was visible. It was evident that this female had long carried about her a tumour in a state of indolence, and that the lancinating pains, which attended and announced its malignant development, were mistaken for peritonitis. M. Laennec sermonises a little on these mistakes, and gives a sly cut or two to the great Broussais for seeing nothing but gastro-enteritis in all diseases.

---

5. *Catalepsy.* The narrators of this case (M. M. Lagarde et Lenormand) inform us that they should have hesitated to publish it, had they alone been the witnesses of it; but, as the patient had been seen by Messrs. Recamier and Laennec, who vouch for its authenticity, they cannot fear to obtain implicit credence as to the statements. There is nothing, indeed, of the incredible in the particulars of this young lady's case. She has not been able to see with her fingers—to predict what was in the womb of fate—to answer questions which were only put in thought—to read sealed letters. or such exploits as have been vouched for on high authority in this country. She has, however, suffered a series of curious and remarkable transitions, from one train of morbid phenomena to another, which are not at all impossible or even improbable. But we shall proceed at once to state the case as briefly as we can.

*Case.* Mademoiselle ——— was born at Lisle in 1809, but lived principally in the country with her parents, to whom she was affectionately attached. Up to the period of her first menstruation, at the age of fifteen, she enjoyed fair health, with the exception of some pains in her stomach and constipation of bowels. She only menstruated thrice, and then they stopped. Early in the month of June, 1284, she was placed in a boarding-school in Paris, where her sister was also resident. Here she became completely nostalgic—pining for a return to her parents, and to the delights of a rural life. She, however, pined in secret, lest her situation should render her parents unhappy. On the 29th November, of the same year, she was seized with a fever, after six nights passed without sleep. It was treated by the usual means, and she recovered in three weeks. In the course of a few days after recovery from the fever, she became affected with obstinate constipation, and a difficulty in taking any food. Purgative medicines had little or no effect in moving the bowels. Symptoms of chorea now appeared. The head, arms and legs, were in constant motion. She also complained of deep-seated pain in the chest, especially under the sternum. Towards the beginning of January, 1825, the choreiform symptoms began to diminish; but they were succeeded by others of a very different character, namely, trismus, and an almost permanent contraction of the muscles of deglutition. It was now impossible to introduce any aliment into the stomach. During the space of eight days, not more than a few drops of liquid could be got down the throat, although various means were tried for that purpose. A few days after the commencement of this last train of phenomena, the patient became affected, during a portion of each day and night, with cataleptic symptoms. These were as follows:—complete immobility—rigidity of the whole body—eyelids shut and fixed—eyes turned up—pupils natural, as was the expression of the whole figure—pulse feeble—skin alternately hot and cold—general and complete abolition of all the senses, except that of hearing. The patient could hear every thing that was said around her, but could make no answer or sign. On recovering from these states, she complained of having had, and of still feeling, a violent pain under the sternum. In the intervals she condoled with her parents, and did all in her power to second the means which were used for introducing aliment into the stomach. Cold affusions and various other remedies were tried, but with little or no effect. In the course of the third week of the trismal and cataleptic attack, some changes began to take place. She could no longer hear distinctly what was passing around her—her limbs became moveable—and, at length, they assumed readily any position in which they were placed by the attendants, thus the disease was now complete catalepsy. These attacks were, for some days, exactly periodical. They began at four o'clock in the afternoon, and lasted till half-past ten in the evening. The intervals were passed in much pain and anguish about the stomach, greatly augmented by the introduction of any aliment whatever.

On the 14th of January she was conducted to the hotel of her parents, whither she was very anxious to go; but the catalepsy came on there, also, at the usual hour. Various means were tried to dispel the attack, but the more it was interrupted, the more it was protracted, and the greater were the patient's sufferings. She had now arrived at the ninth week from the beginning of her illness. On the 27th January, during one of the cataleptic paroxysms, she was seized with certain convulsive movements. Her arms were suddenly thrust out of the bed —her countenance expressed great suffering—her eyes suddenly opened, and were convulsively turned upwards—the lower jaw descended and closed alternately—she frothed at the mouth—and, in a few minutes afterwards, began to sing! In the course of the night, the choreal affection of the head returned. The paroxysm ceased; but returned, and went through the same course, on the 28th and 29th. During the succeeding days, a kind of somnambulism took place. The patient sung several favourite airs—talked aloud—and went through several movements during the cataleptic period, which, however, was shortened in duration, and was succeeded by a complete oblivion of all that occurred during its continuance. An antimonial plaster, which was applied at this time to the epigastrium, caused dreadful sufferings for eight or ten days, but not the slightest interruption of the catalepsy and somnambulism. By the 15th February, her state of exhaustion and discouragement had arrived at its utmost degree. She was scarcely able to utter a word—appeared indifferent to surrounding objects—scarcely took any nourishment—and, during the spasms, was in great terror. About this time, the cataleptic accession was preceded by a sense of excessive coldness over the whole body, although the skin felt of the natural temperature to others. She had also most obstinate constipation. From the 19th February till the 10th March, the catalepsy so much increased that there were now but very short intervals between the paroxysms. She was like a figure of wax, which could be moulded to any form, and would remain in such form till altered to another. The want of nourishment had induced an extreme degree of emaciation, and her pulse was remarkably feeble. She went a fortnight or three weeks at a time without any alvine evacuation, and was sometimes thrown into convulsions when a motion was procured by enemata. The fæces were always unnatural. About this time some suspicions were entertained by M. Recamier, and also by Laennec, that the young lady dissembled a little. Thus, it was distinctly proved that, when she thought she was quite alone and unperceived, she could make use of her hands very freely, but, the moment that any one approached, she was cataleptic from head to foot. There can be no doubt, indeed, that young women will sometimes feign states and conditions (and apparently without object or end) that would seem almost incredible—nay, impossible;—but such is the nature of woman! From the 13th March, the catalepsy evidently and daily declined, and the young lady began to take nourishment, move, speak, laugh, &c. The paroxysms became slight, and most regularly periodical. This periodicity did not deviate a moment from its fixed periods till the 30th March. She passed large quantities of urine, some of it limpid, other portions of a natural colour. The emaciation now diminished sensibly, and the young lady began to assume an air of comparative gaiety. With the exception of a few nervous and anomalous attacks, the patient went on progressively to convalescence, and, on the fourth of May, left Paris for the country, completely recovered. The menses had not reappeared.

The authors of this case have appended some pages of reflexions, but these we shall pass over very slightly. We agree with them that, in this case, the separation from parents, who were probably over-indulgent—the great change of life, on coming to the vortex of Paris from the fresh air and rural exercises of her native residence—the nostalgia that succeeded—the derangement of the digestive organs, as evinced by obstinate constipation and epigastric pain, were the first deviations from health, and led the way to the strange and anomalous phenomena which followed. But our authors take no note of the proofs which were afforded, in the course of the disease, that this young lady had an inclination to dissemble—or, at least, to exaggerate the symptoms of the catalepsy. We cannot help thinking, indeed, that most of the phenomena above detailed were modified by the WILL of the patient, and, therefore, that the whole narrative must be taken *cum grano salis*. Still there were many morbid actions of a singular nature, in this case, which could not be raised by the will, and which are curious instances of the mysterious agency of the nervous system.—*Revue Med. Juillet.*

---

6. *Memoir on the "Compact Œdema," or "Induration of the Cellular Membrane of newly born Children."* By M. LEGER, M.D. [*Archives Generales Janvier*, 1825.]

It is now eight or nine years since, in an early number of this Journal, (monthly series) we called the attention of our brethren to the curious and not very uncommon disease now under consideration. Since that period, little has been published on the complaint, and the subject has escaped our notice. The records of medicine are supposed to contain no specification of this disease previous to the year 1718, when Umbezius states a case of it. A child was born about the end of the eighth month, so cold and so indurated that it felt like a piece of ice. The author attributes the phenomenon to the habit of frequenting churches, where the sight of so many statues and images might make an impression on the imagination of the pregnant female! In 1785, M. Doublet describes a swelling of the cellular membrane of new-born infants, hard and without elasticity, which often destroys those tender subjects. He attributes it to a syphilitic taint—an idea adopted by German physicians, but denied by M. Andry, who read a memoir on the disease before the Royal Society of Medicine, in 1787. This society proposed the subject as a prize-essay, and the Memoirs of Auvity in France, and Hulme in England, were crowned with the medal, in 1789. Our countryman attributed the disease to inflammation of the lungs, and does not advert to the great reduction of animal temperature accompanying it—a phenomenon, on which the theory of M. Auvity is entirely founded. This gentleman considers the disease as "a congelation of the adipose substance, occasioned by cold"—a theory embraced by Went, Heake, Goelis and others. The present author (M. Leger) founds his memoir on the careful dissection of more than 150 children who fell victims to the malady, principally at "L'HOSPICE DES

Enfans Trouvés." Previously to giving a succinct description of the symptoms and *post mortem* appearances, he relates three cases, as specimens. Of these we shall present the principal particulars to our readers.

*Case* 1. A. G. born the night before, was received into the hospital on the 10th March, 1823. It was feeble and small, yet its limbs and face were sufficiently fleshy. The temperature of the body might be said to be cold rather than warm to the touch. The skin was of a reddish colour throughout, especially the extremities. It cried little, and was stiff in all its motions. The following day it was taken to the infirmary of the Hospice, presenting a yellowish red colour on the surface. The temperature was still lower than on the preceding day. The legs were sensibly indurated. The infant appeared in a constant stupor, its eyes fixed, and the eyelids somewhat open, its countenance presenting, occasionally, the expression of pain; voice extremely feeble. On the third day the cellular induration had invaded the whole of the lower extremities, and the fore-arms. It had ceased to cry—the colour was of a red violet appearance. On the fourth day, the induration was extreme. It died the same evening.

*Case* 2. E. Jasline, was received in the Hospice on the 16th February, 1823, being born on the same day. It was taken into the Infirmary on the 17th. It was observed to be small and delicate, though the flesh was sufficiently abundant. The members and face were much indurated, and devoid of elasticity. The temperature was very low—the colour of the skin was of a blueish red, becoming yellow when pressed by the finger. It cried not—appeared tranquil—kept its eyes shut—and seemed in a state of stupor. It died the same night.

*Case* 3. R. Alexis, was born on the 17th April, 1823, and received into the infirmary of the Hospice on the 19th, for a slight ophthalmia. Its flesh was firm, well developed, but without induration—voice strong—skin reddish coloured—temperature natural—in short, the infant appeared perfectly well. The following day its face was of a violet hue, and the cheeks, on pressure, felt cold and somewhat indurated. Its voice was weaker this day. On examination, it was observed that the chest was unnaturally compressed by the tightness of the swaddling-clothes. The feet and fore-arms were cold, but very little hardened. The child was ordered to be left unbandaged. On the succeeding day, the temperature was natural—the induration of the cheeks was gone—the strength of the voice was restored; but there was a yellowish tint of skin. On the day after this, the infant was again tightly swaddled with bandages over the whole body—the induration of the cheeks had returned—the loss of temperature was considerable—the voice enfeebled—the motions of the limbs embarrassed. On the 5th day from its reception in the Hospice, notwithstanding the *recommendations* to the contrary, the child was still kept tightly bandaged, from head to foot!

The induration had made progress—the voice was extremely feeble—the temperature still farther reduced—the yellowish and violet tint universal. The child died in the evening !*

Our author now proceeds to an enumeration of the appearances, post mortem, in this curious disease, as deduced from a great number of dissections.

1*mo. Aspect.* The body is generally below the middle size—the tint of the skin is a mixture of yellow and violet, varying according to the intensity and duration of the disease. The abdomen is of its natural softness. The induration is particularly observable in the outside of the legs and soles of the feet, and in the corresponding parts of the upper extremities. The cheeks are often the most indurated parts, being shining and salient. The lips are generally of a deep red colour—the upper eyelid is projecting. The trunk pretty frequently partakes with the extremities in hardness, but not in so great a degree. When the indurated integuments are cut into, a yellowish serum oozes out, sometimes copiously, after the scalpel, sometimes scantily. The adipose substance presents a mass of small granulations, yellowish and dense.

2. *Respiratory System.* It is here, and consequently in the circulatory system, that the most serious lesions are to be found. The pharynx is often contracted from the serous infiltration above-mentioned, and the lining membrane sometimes redder than usual. The glottis and epiglottis partake in the same ; hence, without doubt, the feebleness of the voice. But what is most remarkable, and never absent, is the congestion of black blood in the lungs, which renders them dense, heavy, of a livid colour, and marbled, so as to closely resemble the substance of the liver. They do not crepitate between the fingers, and they sink in water. This state is sometimes partial, sometimes almost general. There is, for the most part, some effusion of yellowish serum in the bags of the pleura.

3. *The Circulation.* The pericardium generally contains much serosity, the membrane itself being almost always sound. The heart is constantly found of enlarged volume, its parietes being thickened and condensed—sometimes softened and flabby. The cavities are usually gorged with blood in clots. The internal surface of the large vessels is often found of a yellowish or reddish colour. The veins are filled with black blood.

4. *Organs of Digestion.* The mucous membrane of the intestines

---

* It is astonishing that at this time of day such a barbarous custom should be tolerated in a public institution, as that of bandaging an infant, from head to foot, the moment it is born. Talk of the bed of Procrastes ! this is decidedly a more cruel practice. It is probable that the greater frequency of this complaint in France than in England, may be partly owing to this custom.—*Ed.*

sometimes presents marks of phlogosis. The cavity of the peritoneum generally contains some fluid.

5. *Nervous System.* The state of stupor which usually marks this disease, would lead one to expect unequivocal lesions in the cerebro-spinal system—but these are rarely found. Sometimes a little serum is seen in the ventricles of the brain, and sometimes the vessels are rather turgid with blood.

Such are the organic changes found after death in this curious disease. In respect to the chemical qualities of the fluid infiltrated in the cellular membrane, they are precisely the same as those of the serum of the blood in the same subjects. One remarkable quality of this serum is, that after standing, for some time, it coagulates and takes the shape of whatever vessel it is in. On pressure, this mass separates into a membranous and fluid portion.

We need not follow our author in his refutation of the hypotheses, rather than the theories which have been formed respecting this disease. His own idea is, that some mechanical obstruction to the circulation exists, and that a serous effusion is the consequence. The serum having the property of coagulation, the œdema is hard instead of being soft. But what is the cause of the coagulating quality in the serum of the blood, he cannot tell.

The disease is often, but not always mortal. Every thing that can enliven and promote the free circulation of the blood, is most calculated to remedy the disease.

---

7. *Ulcer on the Surface of the Brain.* John T. Gomdry, a chasseur in the 7th Regiment of Infantry, experienced, all at once, on going down to guard, an acute pain over the eyebrow, which he in vain tried to dissipate by the application of cold lotions and other means. In the course of the following day, some symptoms of gastro-intestinal irritation came on—the tongue became loaded—the appetite failed—and the patient felt extremely weak. Three days after this he was sent to the Val de Grace Hospital, under the care of M. Gomdry. The phenomena of gastro-intestinal inflammation were then unequivocal, and 30 leeches were ordered to the epigastrium, his diet being of the most rigid kind. By these means the gastric affection was checked, but the cephalalgia remained as severe as ever. Twenty leeches were, therefore, applied to the sides of the neck, which mitigated the head-ache, but did not entirely remove it. The patient was able to take food, and to walk about. All at once, however, and without any apparent cause, the supra-orbital pain suddenly returned, and with greater violence than ever. The patient was forced to cry out, and endeavoured to ease the pain by strong compression of the head. More leeches were applied, and also cold applications to the head, with sinapisms to the lower extremities; but these means procured no relief. The phenomena of gastro-intestinal inflammation recurred with increased intensity—the tongue became very red—the thirst urgent—pulse hard, full and frequent. During the next eight days there was no change for the better. He suffered dreadfully from his head—frequently cried out with pain—and was obliged to keep his eyes shut to avoid the light. In this period, leeches, cold lotions, blisters, and various means, were used without effect. At length, irregularity of the pulse and subsultus tendinum came on, with excessive heat of skin, and the patient expired on the 21st September, 26 days after his entrance into the hospital.

*Dissection.* There was some inflammation of the mucous membrane of the stomach, particularly about the cardiac orifice. There were also traces of phlogosis throughout the whole track of the small intestines, and there were small ulcerations in the ileum. At some inches from the valve of the colon there was a digitiform appendix of the intestine, which extended some distance, and became attached to the parietes of the abdomen.* On the under surface of the anterior lobe of the right hemisphere was an ulcer, thirteen lines in length by seven in breadth, having a yellowish surface with ragged edges. The subjacent cerebral substance was sound. The portion of meninges, corresponding to this ulcer, was destroyed. The rest of the brain was unaltered. The tunica arachnoides was inflamed throughout. There was nothing else particular in the other viscera.

There are but few cases on record of distinct and primitive ulceration of the brain, and we fear there are no certain marks by which we can recognise their existence in the living body. The severity of the symptoms in the present case would have led any one to expect some severe organic change in the brain or its membranes.

P. S. In the same Journal, another case of ulcer of the brain is related by Dr. Scoutteten. The patient was a soldier, 24 years of age, who had been condemned, as a punishment, to some extra labour, at which he had been several times taken ill. He was sent to the Military Hospital at Metz, where he was seen, on the 1st August, by Dr. Moizin. His skin was then of a yellowish tint—his nostrils swelled and ulcerated—tongue pale—pulse feeble and slow—temperature below par—some cough—appetite natural. Three days before his death there appeared an œdematous swelling of the forehead and eyelids, with head-ache, but not very severe. On the next morning, the tongue was remarked to be red and dry, the thirst ardent, the skin hot, the pulse quick and hard, delirium—sickness at stomach, and coma. From the appearance of the gastro-enteritis the treatment was antiphlogistic.

*Dissection.* The viscera of the chest were sound. The mucous membrane of the stomach was inflamed (chronically) throughout its whole extent. In one place there was a large patch of acute inflam-

* We suspect that these appendices sometimes play an important part in pathology, and tend not a little to puzzle the practitioner, who seldom suspects that there can be any thing but a regular canal from the stomach to the anus. But more of this in another place.

mation. The small intestines were but little affected, but the internal surface of the colon presented marks of chronic inflammation throughout its whole extent, with numerous ulcerations, but not deep. There was an immense quantity of yellowish bilious mucus in all the intestines, among which were numerous worms. On the posterior lobe of one of the hemispheres there were two small ulcers, which went no deeper than the cortical substance of the brain. One of them was six lines in length, with a foul surface. Around these ulcerations, the brain and its membranes were much injected with blood. The rest of the brain and the cerebellum were perfectly sound. There was some inflammation of the tunica arachnoidea.

Here we see nearly similar lesions of structure, while the living phenomena were as different as black is from white! What encouragement this for the symptomatologist!

---

8. *Hospital Reports—Hotel Dieu.** Two hundred and five patients were received by M. Recamier, during the quarter, of whom 36 died—or one in six. This is a large ratio of mortality among the miscellaneous inhabitants of an hospital! Of the diseases, 175 were acute, and 30 chronic. The mortality among the *latter* was greater in proportion than among the *former*. Of the acute diseases, pulmonary inflammation constituted the greater portion, occasioned evidently by the wide range of atmospherical transitions. Next to pulmonic, were gastric affections; and, in the third rank, were the eruptive fevers, especially variola. Lastly, rheumatic, peritoneal, and erysipelatous inflammations closed the ranks.

1. *Intermittent Fevers.* Several patients contracted this disease in Paris—others had been lately in marshy districts. M. Recamier allowed *several paroxysms* to take place, without exhibiting any medicine. He then administered an emetic of ipecacuan, or a purgative of salts and antimony, which, in several instances stopped the ague. In other cases, the disease went on for a certain length of time, and ultimately gave way to diluents and evacuants. In only one case was the bark required. Now this is a very pleasant illustration of the "*medecine expectante,*" exhibited by M. Recamier to his pupils. But to the poor patients, who have to drag through so many agueish paroxysms, without medicinal aid, the experiment is no joke! We only wish that M. Recamier had personal experience of the horrors of an ague fit—we are convinced that he would soon prefer bark or arsenic to the visits of the fenny fiend! We know, indeed, that ague will, in general, wear itself out in a sound constitution; but is it certain that the constitution receives no detriment by such a process? We are inclined to think—nay, we have the strongest reason to know, that it seldom can sustain the repeated shocks

---

* M. Martinet's report of M. Recamier's practice, for the second quarter of 1825.

of an ague, without injury to some organ. There are few individuals who have not some weak point—in body as well as mind: and this weak point (in body) is almost sure to suffer by the violent oscillations of an intermittent fever. But, allowing that no ultimate derangement of function or structure succeeded this protracted cure by the "*medecine expectante*," we do maintain, and that from the best source of knowledge, personal experience, that the "methode expectante" is a cruel and unnecessary exposure of a fellow creature to "corporal sufferance," which it would be unjustifiable to inflict on "the beetle which we tread on." It is in direct violation of the very first principle of medical science—the mitigation of human pain. Who, but a marble-hearted philosopher, could look calmly on the repeated paroxysms of an ague, and say, "let the disease wear itself out," when he had the safe and salutary means of checking at once its baleful career? We hesitate not to tell Professor Recamier that he acts the part of a cruel tyrant—and that under the reign of Dionysius, Nero, or Heliogabalus, he would have been promoted for the ingenuity with which he inflicts his tortures on suffering humanity! We quote the following passage as an eternal disgrace to medicine.

"M. Recamier voulait *montrer aux élèves qu' il n'est pas néçessaire de tourmenter les malades par des medicamens ou des evacuations sanguines, pour amener a bien des affections dont l'economie se debarrasse d'elle-même, lorsque l'epoque de leur cessation naturelle est arrivée.*"

What is medicine designed for, if not to curtail or cure diseases? Why does M. Recamier take men into an hospital—if it is only to shew his pupils that Nature can cure diseases without the aid of the physician? We hope this philanthropic professor may soon be visited by an intermittent fever! He will then learn to appreciate the penalty of disease, and hasten to mitigate it by the means which Providence has put into his hands! —We will not be accused of acrimony in these remarks. We speak from personal feeling, and we wish to lessen the sum-total of human sufferings—not to look on, indifferent spectators of a fellow-creature's woe. From an expression or two in the above passage, we suspect that M. Recamier (who is known to be hostile to the doctrine of Broussais) had something more in view than immediately meets the eye. He probably wished to shew his pupils that the "*gastro-enterite*" of his contemporary did not require to be "*tormented with* medicines and leeches"—though we think a far better way of combating the theory of Broussais, would have been to cure this gastro-enterite in two or three days by a *tonic*. Be this as it may, we hope the Professor, whom we hold in great respect upon all other occasions, will not repeat his experiments at the expence of his suffering patients again.

2. *Arachnitis.—Case.* J. Larcheveque, 47 years of age, of athletic constitution, had long been a prey to moral affections of a melancholy nature. For five months he had been subject to pains in the head, which lately became violent and almost continual. To these were added pains in his loins, with much difficulty in walking. For three months he had used the hot baths at the St. Louis Hospital without benefit, and on the 26th May, 1825, he entered the Hotel Dieu. His intellectual faculties were now considerably impaired—his answers to questions being short and inconclusive. No fever, nor heat of skin—wandering kind of delirium in the night, with such restlessness as required the straight-waistcoat. 27th. Could make no intelligible answers—eyes open and fixed—pupils not dilated—countenance natural—tongue moist—constipation—no complaint of pain—no febrile reaction whatever. In the evening, complete loss of speech and sense. 28th. Stupor—abolition of the intellectual faculties—flexion and contraction of the upper extremities—stiffness of the muscles of the neck on the left side—pulse slow—heat natural. *Twenty leeches to the temples and behind the ears—cold to the head,* by which the stupor was removed, and the sensibility restored. 29th. The stupor again present. *Twelve grains of tartar emetic were given this day in a six-ounce mixture.* 30th. Complete paralysis of the upper extremities—slight febrile reaction—retention of urine. *Eighteen grains of the ant. tart. in a mixture this day.* Cold affusion on the head. He gave some signs of intelligence this day. 31st. Face flushed—pulse irregular—respiration stertorous—pupils dilated. Died in the evening.

*Dissection.* The pia mater and tunica arachnoidea covering the hemispheres above and below, were opaque, very much thickened, and presented many patches of a grumous and albuminous kind. The cerebrum, the cerebellum, and the medulla oblongata, examined with the greatest care, presented not the slightest trace of alteration or of sanguineous congestion. The dura mater of the cauda equina was very much thickened, and covered on its internal surface with a purulent matter. On its external surface were seen several small inflamed tubercles, and two or three small cysts filled with a puriform liquid. The lungs contained a great number of miliary tubercles, and towards their upper part some excavations. There was no disease of any consequence in the other thoracic or abdominal viscera.

3. *Hæmoptysis.* The great heat in the month of June, brought into the hospital several cases of hæmoptysis. On the 18th, three cases came in together, and M. Recamier determined to shew his pupils the effects of large doses of nitre, in this complaint, as employed by the Italian physicians. To each of the patients, therefore, he gave half an ounce of nitre, dissolved in a mucilaginous mixture, to be taken in the course of the day. In one patient, who had been bringing up blood freely for four days previously, and who had taken no other medicine, the hæmoptysis was completely arrested during the first day in the hospital. The day after, it returned, and was again stopped by the same medicine, and did not afterwards recur. This patient took the half ounce of nitre in the course of four hours, by which the urine was very much increased, and some disagreeable sensations were produced in the stomach and mouth, but no other effects. In the second case, bleeding had been previously but ineffectually employed, and the hæmoptysis continued abundant. On the second day of the administration of the nitre no trace of blood was perceptible in the expectoration. The medicine was continued for two days more, as a precautionary measure. In this patient, no inconvenience to the stomach was produced by the nitre. The third case was that of a man, 45 years of age, who had been subject to severe hæmoptysis for ten years previously, the attacks recurring about once in two years, and generally giving way to blood-letting and leeches to the anus. The hæmoptysis had this time continued three days, accompanied by great embarrassment of the breathing, and a crepitous rattling in the lower part of the left side of the thorax. He had been bled several times before he entered the hospital, both locally and generally, but the hæmoptysis continued. Like the two other patients, he took the nitrous mixture. The second day he felt much less enfeebled—the expectoration was not so bloody. The treatment was continued, and the expectoration soon became untinged with blood, and puriform. Ultimately, however, the patient sunk with regular hectic fever, presenting, on dissection, several tubercular excavations in both lungs.

We have certainly seen good effects from nitre in considerable doses, given in the common infusion of roses, well acidulated, in conjunction with laudanum, in hæmoptysis ; but we never gave it in such doses as above, nor have we seen such doses exhibited by others. The remedy is worth trial.

4. *Pulmonic Inflammations.* M. Recamier follows the English method of decisive depletion in these phlogoses, and with much success. We need not, therefore, dwell on this part of the report. In the course of one fortnight two cases of the gangrene of the lungs (a rare disease) presented themselves in the hospital. One of the patients had small-pox, complicated with pulmonic inflammation. The other died on the 25th day from the commencement of the pneumonia, and presented gangrene of the lungs.

5. *Eruptive Fevers.* Small-pox made considerable havoc in the hospital this quarter—and, unfortunately, was contracted there, in most instances, when men were convalescent from other diseases—the wards having, in fact, become a focus of infection. Five died during the quarter. The trachea and larynx were found considerably inflamed. The violet redness (la rougeur violacée) extended to all the bronchial ramifications. The lungs were generally in a state of engorgement.

